# Microbiome Yarns: microbiome of the built environment, paranormal microbiology, and the power of single cell genomics^1^,^2^,^3^,^4^

**DOI:** 10.1111/1751-7915.13274

**Published:** 2018-05-16

**Authors:** Kenneth Timmis, Franziska Jebok, Manfred Rohde, Gabriella Molinari

**Affiliations:** ^1^ Institute of Microbiology Technical University Braunschweig Braunschweig Germany; ^2^ Institute for Educational Science University of Freiburg Freiburg Germany; ^3^ Central Facility for Microscopy Helmholtz Centre for Infection Research Braunschweig Germany

## Part 1

*BBZ, Studio 7A, BBZ Plaza, Burbank, 7.30 pm*.

*Abigail Repor‐Tastory, Discovery Presenter, turns to face the camera*: Good evening and welcome to a new episode of ‘Discoveries that Change our Lives’. Our guest this evening is once again Dr. Anastasia Noitall‐Most^5^ from the Streber Elite University of Los Angeles. Good evening Dr. Noital‐Most *(shaking hands)* and thank you for appearing on the program.

*Dr. Noitall‐Most:* Good evening Abi, it is always a pleasure to be here.

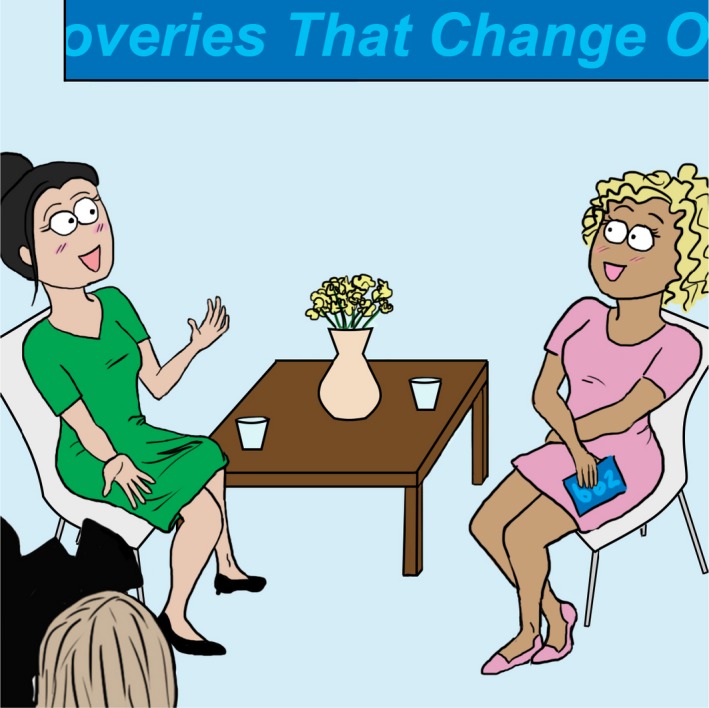

*Ms. Repor‐Tastory:* Ani, this evening we want to discuss what seem to be amazing discoveries about the home microbiome and … did you mention ghosts in our pre‐show chat?

*Dr. Noitall‐Most: *Yes, Abi. Some quite unusual and highly controversial discoveries have been reported recently. The backdrop to these new findings is that the microbial communities present in places we spend any time in – our homes, workplaces, garden sheds, tattoo studios, super yachts, karaoke bars, gyms, restaurants, tanguería, beauty parlours, etc., the so‐called *Built Environment* – contain the microbes of our skin^6^.

*Ms. Repor‐Tastory:* Yes, there has been quite a bit of news on the topic, especially via social media. But why is this so newsworthy?

*Dr. Noitall‐Most:* Well, previously, it was thought that air had a sort of nondescript air‐dust characteristic microbiota derived from wind‐, garden blower‐, road sweeper‐, jet engine‐ and traffic‐suspended microbes from soil, vegetation, inanimate surfaces, and even dust from the Sahara, which is regularly blown north from Africa^7^, and that inside air is just outside air let in through doors and windows. Augmented, of course, by all the amazing stuff dogs bring in, mostly but not only on their paws, and broadcast into the air when they perform their scratching frenzies to entertain us. And by the microbiomes of our wonderful zoos of smaller, more discrete, house guests, like silverfish, dust mites, carpet beetles, cockroaches, bedbugs, *et al*, who relish the varied tasty treats we thoughtfully provide for them in our built environment, and who are launched into the air by the daily/weekly/monthly clean‐up. And, occasionally, in some less well‐ventilated buildings, by a misma of fungal spores.

*Ms. Repor‐Tastory, signalling intense disgust:* Ughhh, the thought of those creepy‐crawlies being part of my home makes me shiver!

*Dr. Noitall‐Most:* Just so! Anyway, the key thing is that metagenomic analyses have shown that, although air of the built environment contains soil‐vegetation‐road‐Sahara dust microbes, it also includes significant numbers of microbes derived from human (and, for households with pets, animal) skin^6^. As you know from social media, we humans are 50% microbial, that is our bodies consist of about the same number of microbes as human cells or about 40 trillion of each^8^. Now, if we imagine that these 40t microbes might reproduce every few hours, it is pretty obvious that we would explode and become unceremoniously buried under tons of microbial slime pretty sharply if we did not get rid of them at the same rate. Which we do: most of our microbial friends live in our colon, and those that have outstayed their welcome are unceremoniously dispatched, mostly daily, to wastewater treatment plants for job retraining and the adventure of a new life. However, the number of cells not sanitarily disposed of in this manner, those covering hair, skin, airways, etc., is still pretty large and these are simply jettisoned into the environment, mostly our built environment. And, crucially, since individual humans have distinctive microbiomes, we thereby personalize with our own microbes the surfaces and dust in rooms we enter/occupy^6^.

*Ms. Repor‐Tastory:* Ok, but what is the significance of this – how does it affect viewers?

*Dr. Noitall‐Most: *Well, the thing is that even a relatively transient sojourn in a room leaves a microbial trail – we all shed skin continuously: the average human sheds on average 30 million bacteria per hour^8^ – more in the case of blokes that dry shave – so, for example, if an uninvited guest pays a call while we are at work, on a furtive romantic assignation, out Morris Dancing, placing a sure‐fire bet on a pony, or borrowing sugar from the neighbour, he/she will unwittingly leave their microbial calling card. So: the microbiota of the built environment can be used for criminal forensics^9^.

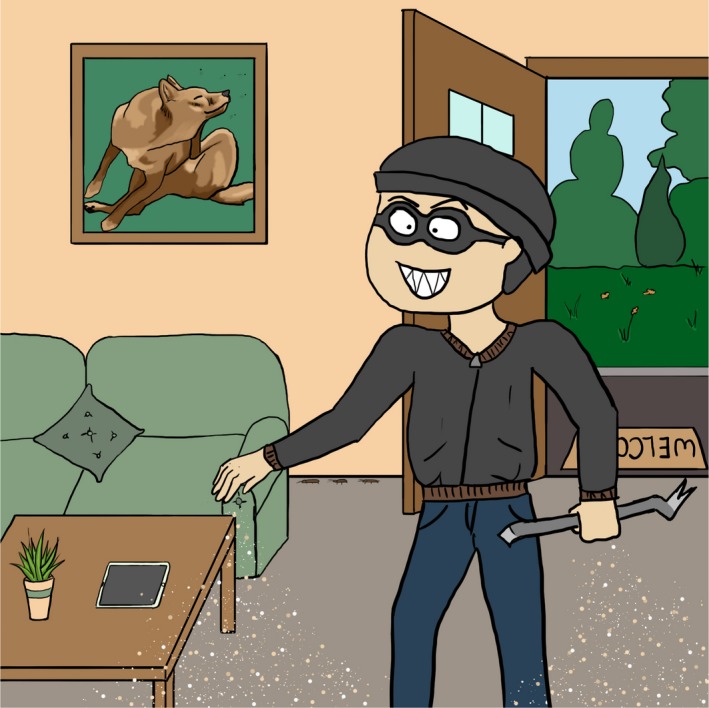

*Ms. Repor‐Tastory:* But doesn't the wonderful DNA profiling technique already do the job?

*Dr. Noitall‐Most:* Yes, Abi: it certainly does. However, DNA profiling has recently been getting a bit of bad press^10^, not because the technology itself is problematic, but because its implementation is not always perfect. So, to have additional methods and lines of evidence to identify villains invading and plundering our personal space is becoming essential. And moreover: given the fact that humans spend most of their time in the built environment, merrily breathing in all of its microbial bounty day and night, its potential influence on health and disease is coming under increasing scrutiny^11^.

*Ms. Repor‐Tastory:* I see: so the microbiome of the built environment is a really important thing right now. But what is the issue with ghosts?

*Dr. Noitall‐Most:* Well, as you said, this story was also widely spread by social media and one individual who learned about it this way was Professor Humpfrey Geisterbahn of the Department of Paranormal Studies at the University of Eastern Carpathia, who immediately had the idea that perhaps ghosts have a microbiome and may leave a signature microbial trail when they go on their nightly constitutionals. After consultation of his funding agency, and the BPRD^12^, he initiated a major new research programme based on this idea. Being a field mycologist by training, he then designed a rather neat experiment.

First of all, he identified several well‐characterized ghost paths, all very dark corridors, remote from windows, in five uninhabited houses older than 200 years. He then designed special microbiome traps – basically 30 × 30 cm moist pads containing a sticky resuscitation‐nutrient solution, arranged in pairs, one above the other, 80 cm and 140 cm from the floor – and placed them along the paths at 2 m intervals. As controls, he placed traps along stretches of corridor not documented to be ghost flight paths. Over a period of several weeks, he installed himself in each house at an inconspicuous location where he could observe in comfort and, after every passage of a ghost, removed the microbiome traps for analysis, and replaced them with new ones for the next passage. Where possible, three repeat traverses of each ghost were made in the study, since some biologists believe 3 replicates to be the magic number for statistical analysis, though not all ghosts were always compliant and occasionally took irregular routes. Somewhat frustratingly, one ghost always took evasive action when confronted with a trap.

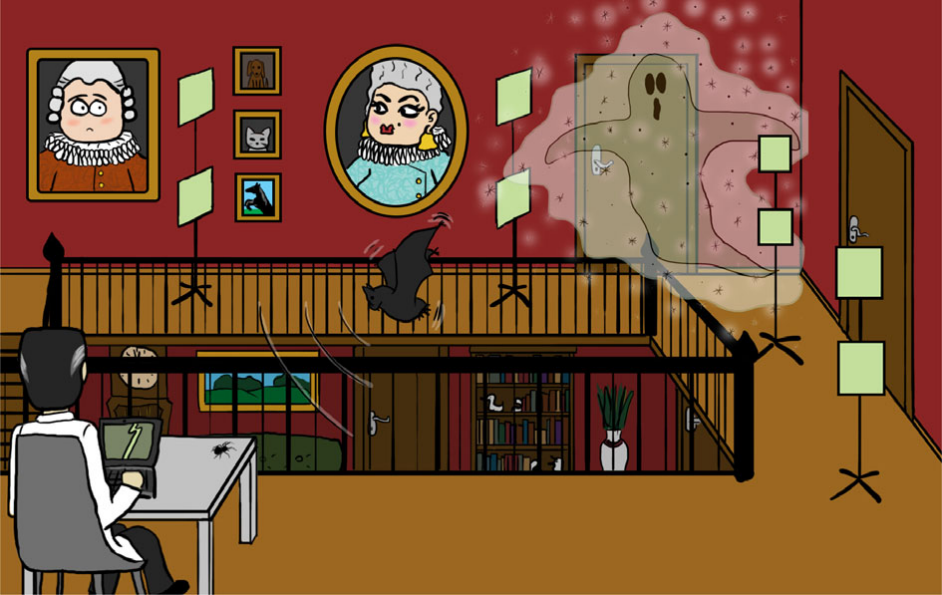

*Ms. Repor‐Tastory, shifting uncomfortably in her chair:* OOOOHH! Gosh, Ani. This is so spooky! What was the outcome of the experiment?

*Dr. Noitall‐Most:* Yes, it is certainly not your everyday grad student research project fodder! Anyway, the microbiome traps were subsequently placed in closed containers, and transported to a high security biosafety facility, an upgraded version of what had previously been a cold war biological warfare research station. Here they were analysed by cultivation, to obtain and identify the bugs that would grow on lab media, and by metagenomics, to capture all microbes and obtain approximate quantitative estimates of them.

The results of Professor Geisterbahn's experiments were rather interesting: the cultivation experiments revealed only typical well‐known dust bugs in both ghost and control paths, with no detectable differences between the two. However, the metagenomics analysis revealed that, in addition to the normal dust microbes found in old houses, there were a few different bugs that were specifically associated with the ghost paths. All of the ghost‐associated microbes were spore‐forming bacteria and fungi – i.e. microbes that produce spores which are a dormant form that can survive for decades, even centuries, and probably much longer. But, importantly, many were completely new, although a few were distantly related to *Gloamingia shiveri*, a bacterium only found so far on the facial whiskers of vampire bats. And since none of the cultivated microbes were the same as or similar to the ghost‐associated bugs ‐ GAB for short – it seems that GAB are not culturable.

*Ms. Repor‐Tastory, shivering involuntarily:* So there really are ghost bacteria!

*Dr. Noitall‐Most:* Well, at this stage, results were rather preliminary and, as you can imagine, not readily accepted by the microbiology establishment. And, because the new bugs were, well…… new, there was no information on what they might do or have done.

*Ms. Repor‐Tastory:* In other words, they were ephemeral?

*Dr. Noitall‐Most:* Quite! Now, since Professor Geisterbahn had reached a dead‐end, he sought assistance from the Imaging Group of Mabriella Golinari and Ranfredy Mohde of the Walpur Gisnacht Institute for Cellular Pathology in Bad Hurzbarg in Northern Germany, which is not only a world leader in microscopy but also in growing difficult‐to‐culture microbes and exploring their biotechnological potential, for example the production of inhibitors that could be leads for new medicaments^13^. Well, Mabriella and Ranfredy were understandably sceptical of the ghost context but, on the other hand, interested to access new microbial diversity, whatever the source. Moreover, the international impact of their recent work on the bacterial basis of memory^14^ had been recognised by a prestigious award that allowed expansion of their team, so they had enough capacity to take on a new topic.

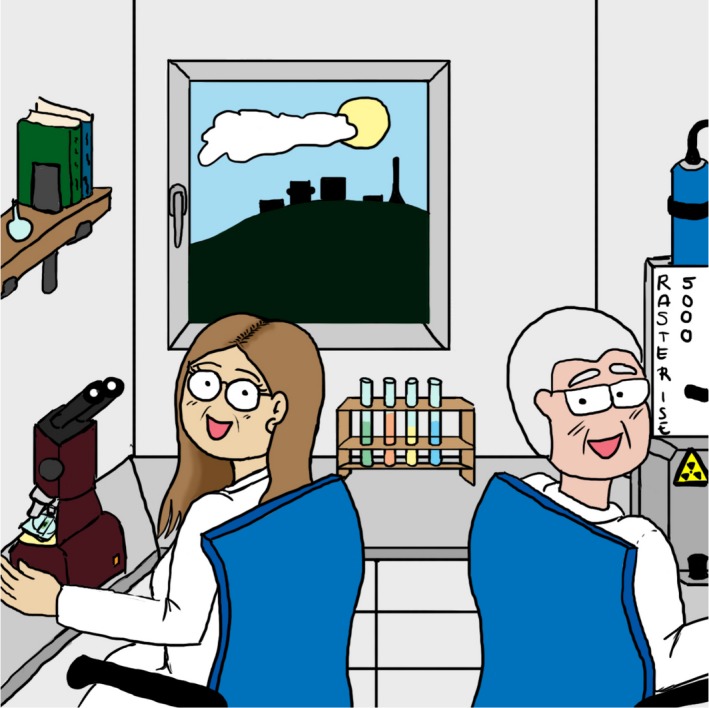

Initial microscopic scrutiny of the material collected on the ghostpads revealed a lot of spores, but also about 40% of vegetative cells, as we can see in the screened image^15^.

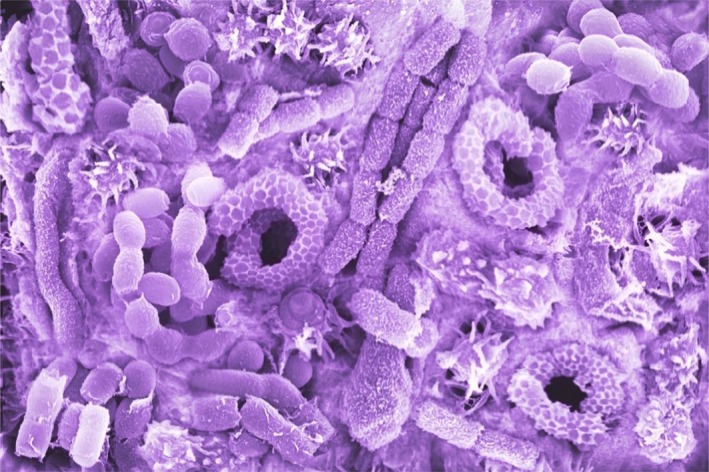

The Bad Hurzbarg team tried out their entire spectrum of resuscitation media with very low nutrient concentrations and looked for germination of spores, but without success. They then brewed up several new media containing dust collected from the burial crypt of a nearby abandoned abbey and one proved to be a winner. This medium, which they designated *Ghost Organism Rescue Medium*, or *GORM* for short, induced germination of practically all spores into vegetative cells, and thus allowed the team to obtain first high‐resolution images of the ghost microbes. Unfortunately, none of the germinated spores went on to reproduce and form colonies.

*Ms. Repor‐Tastory:* Another dead‐end!

*Dr. Noitall‐Most:* Well, not quite! In the old days, it was impossible to study the activities of bugs that could not be persuaded to grow in the laboratory, so we were obliged to just give up and concentrate on those that could. But now, the genome sequences of individual microbial cells can be determined, analysed by clever algorithms, and metabolic models generated that predict potential activities of the sequenced bugs^16^. So, for example, the genetic information for the production of new antibiotics and other drugs by uncultured bacteria associated with marine sponges has been obtained by single cell genomics, opening up the prospect of producing a new generation of drugs that were previously hidden from us^17^.

So Professor Geisterbahn teamed up with a hot‐shot single cell genomics group at the Cajun‐Bayou University of Southern Louisiana, headed by Leonie Broussard and Hawiovi^18^ Longread, and together they isolated individual spores by micromanipulation, determined their genome sequences, and bioinformatically identified the potential functions they possess.

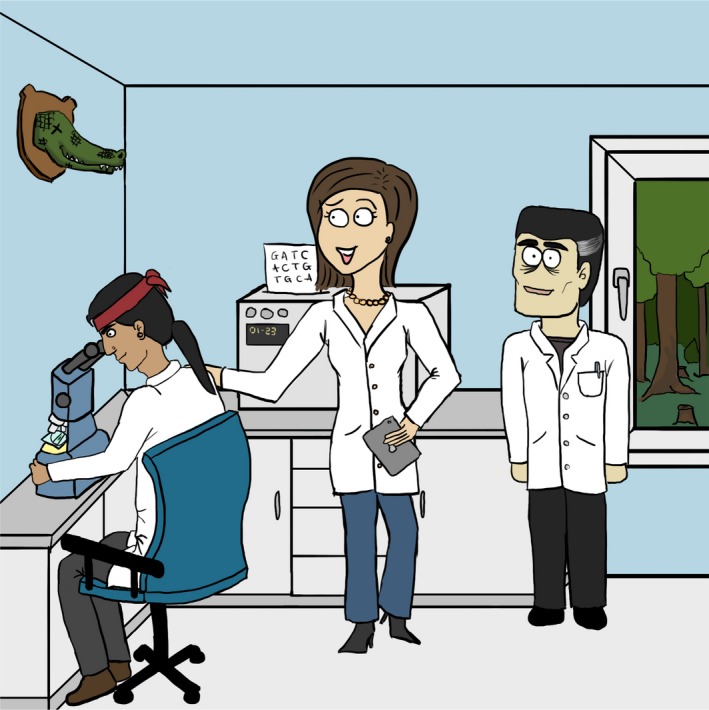

*Ms. Repor‐Tastory:* Wow: not so ephemeral!

*Dr. Noitall‐Most:* Absolutely! And so equipped with the morphology of the vegetative cells, the phylogenetic information given by the genome sequences, and the metabolic features bioinformatically predicted from the metabolic construction analyses, Mabriella and Ranfredy were able to propose proper names for most of the ghostbugs^19^: the bacteria *Wraithia terrifyingi, Poltergeistas bansheea*,* Spookia bogeymana* 20, *Hauntio scariae, Phantomius miragilis*,* Shroudia frighteningi*, and the fungus *Spectormyces creepii*. We can see images of some of these on the screen:

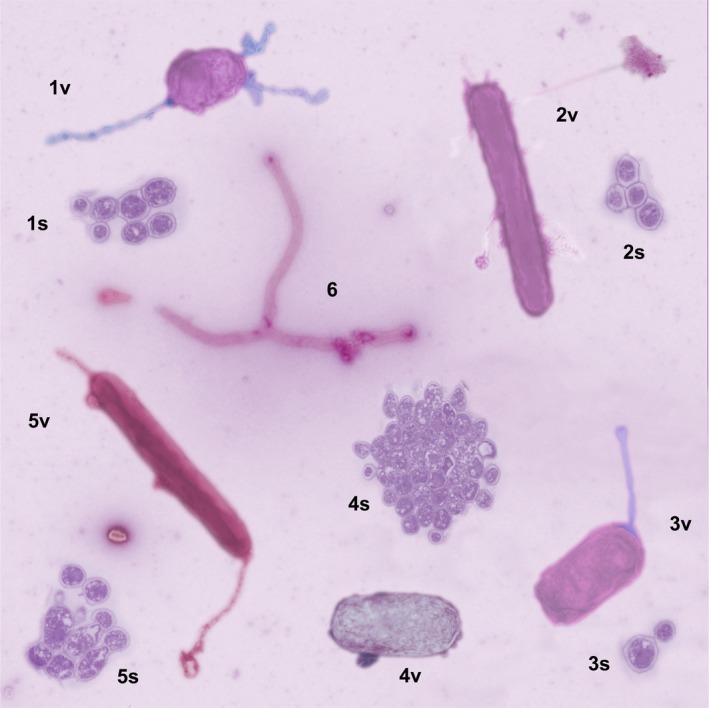

1: *Spookia bogeymana*, 2: *Phantomius miragilis*, 3: *Poltergeistas bansheea*, 4: *Hauntio scariae*, 5: *Shroudia frighteningi*, 6: *Wraithia terrifyingi*. v and s indicate vegetative forms and spores, respectively^21^.

*Ms. Repor‐Tastory, squirming on her chair*: Golly: ghost microbes look rather threatening!

*Dr. Noitall‐Most:* Yes, they have unusual, somewhat sinister cellular forms. Anyway, let's return to the genomic analysis, which is really fascinating. Firstly, all genomes of GAB were fairly small, which was not too surprising, given the fact that GAB probably don't do much most of the time. And: although most of the genomic information deciphered was rather routine, all of the new microbes had several unusual features in common, such as a minimal, low energy – so‐called *Spartan* or scavenger – metabolism, with lots of high affinity uptake systems for nutrients^22^, especially volatile organic compounds, which fits the nutrient‐poor environment they inhabit, and an unusually rich assortment of pathways for compatible solutes, which help microbes survive under dry conditions.

*Ms. Repor‐Tastory:* Ok….do forgive me, but this does not seem super exciting.

*Dr. Noitall‐Most, signalling ever‐so‐slight irritation:* Well, sorry, but not all important scientific results are exciting to the uninitiated!

*Ms. Repor‐Tastory, slightly chastened:* Yes, of course: do please continue.

*Dr. Noitall‐Most:* Hhmmm: well, in addition, the ghostbugs unexpectedly had gene clusters for the production of so‐called gas vesicles, essentially large air bubbles which are normally found in microbes inhabiting salty water bodies, and that allow them to control their buoyancy in order to migrate up and down in order to find environmentally‐optimal conditions for their growth^23^.

*Ms. Repor‐Tastory:* Interesting, but why would GAB make air bubbles?

*Dr. Noitall‐Most:* Well: this is admittedly rather difficult to understand. However, Leonie made some assumptions about the metabolism of GAB, which allowed her to propose that they could make methane and/or ammonia, which are both lighter than air. If the gas vesicles contained either of these instead of air, and if they were airtight, ghostbugs themselves could be lighter than air. Professor Geisterbahn therefore concluded that the gas vesicles in GAB facilitate the floating appearance of their otherworldly hosts on their evening passeggiata. I should, however, remark that the plausibility of this hypothesis has been ridiculed by several experts.

*Ms. Repor‐Tastory, blinking quickly:* Well, I never!

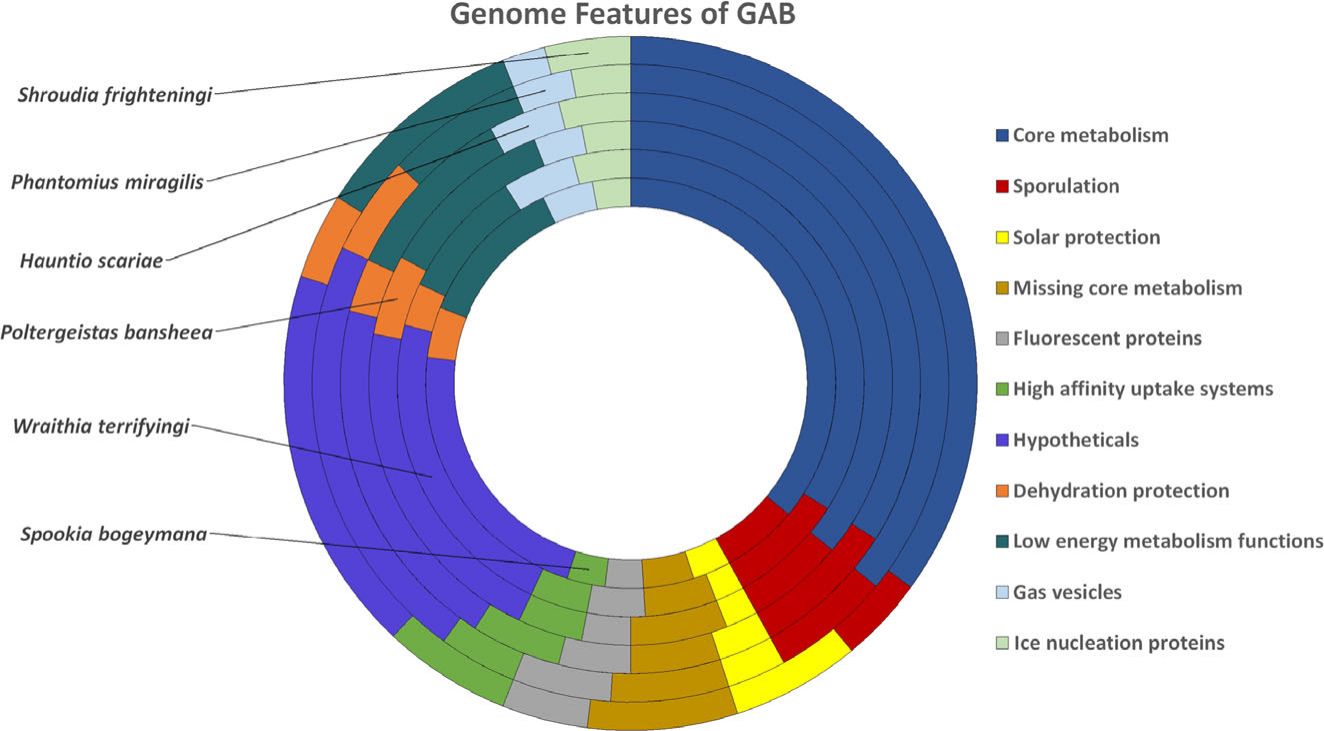

*Dr. Noitall‐Most:* Another unusual characteristic of the genomes was the presence of tandem genes for green, blue and red fluorescent proteins^24^. The archetypal green fluorescent protein, or GFP, was originally discovered in a jellyfish, but a whole range of fluorescent proteins are now known. Interestingly, these proteins have been exploited by biologists for years as molecular beacons and have been extraordinarily useful as tools for visualising and localising all kinds of proteins in cells, and for detecting and measuring gene activity^25^. But the thing is: as everyone knows, when green, blue and red light are combined, white light is the result. So, instead of a jellyfish with a green beacon, we might have a ghost with a ghostly white aura.

*Ms. Repor‐Tastory, hands involuntarily leaping to her mouth:* Oh my goodness: what next?

*Dr. Noitall‐Most, coolly resisting the urge to participate in the drama build‐up:* Another unexpected finding of the genome analysis was a pathway for an unusual form of melanin^26^, which they called Spectremelanin, or SM. As everyone knows, melanin is a pigment found in many organisms, including us, that efficiently absorbs and protects against sunlight and radiation. However, it has different roles in different organisms in different settings, including contributing to virulence of pathogenic microbes^26^. Moreover, right next to the genes for SM were genes for the production of an unusual form of microsporine‐like amino acids^27^, or MLAAs. These also protect against ultraviolet radiation and are known as microbial sunscreens, but have diverse roles in biology and have been assigned to the small group of globally important *molecules of keystone significance* 27. The ghostbug MLAA was designated SpectreMLAA, or SMLAA for short. Naturally, Professor Geisterbahn concluded that, since ghosts almost always appear at night, almost always in dark or low light locations, they must be sensitive to light, especially sunlight, and perhaps UV irradiation. He proposes that SM and SMLAA production by GAB serves the purpose of efficiently mopping up any extraneous sun‐, candle‐ or torch‐light the ghost accidentally may encounter, thereby protecting it from what is probably its primary hazard.

*Ms. Repor‐Tastory, hands covering mouth:* Oh my giddy aunt! So movie depictions of the light‐shyness of otherworldly beings is justified and ghosts do prefer their environments to be as black as Newgate's knocker^27^?

*Dr. Noitall‐Most:* Well, perhaps, but let's not get carried away! And talking about movies: in some popular depictions, ghosts leave behind unpleasant trails of slime ‐ so‐called ectoplasm^28^ ‐ that give us a nasty shock when we touch a door handle, step into some in bare feet, etc. However, the Cajun‐Bayou group did not find genes for the production of extracellular polymers or slime, which is perhaps not surprising, since slime production requires a goodly food supply, which ghostbugs do not generally have.

*Ms. Repor‐Tastory*: So ectoplasm is Hollywood fiction?

*Dr. Noitall‐Most:* Yes, and pretty typical of the film industry. However, the most fascinating finding of the genome scanners was the presence of ice nucleation protein^29^, or INP, genes. These are produced by a number of microbes and have the property of causing water vapour in the atmosphere to form ice crystals on the surface of the bug at low temperatures. The original discovery of bugs that produce INP on plant leaves was an absolutely amazing finding at the time, and early work revealed that they play an important role in the frost damage of crops like strawberries^29^. It has been subsequently suggested that they may play a significant role in climate processes – cloud, rain and snow formation – when swept off vegetation by wind into the atmosphere where they nucleate ice crystal formation^29^.

*Ms. Repor‐Tastory, shifting uncomfortably in her chair:* OOOOHH! And what might the ghostly role of INP be?

*Dr. Noitall‐Most:* Well, Abi: at present, we can only speculate because ghosts are currently not part of the microbiologist's experimental repertoire. But, if we step into the still very much disputed framework of the existence of ghosts and ghost microbiomes …..and if we allow our imagination to roam freely…..and if we recall that people viewing ghosts in cold situations often experience sudden shivery chills on exposed skin, even when their bodies are wrapped up warmly, then we might interpret this as ice crystal‐loaded microbes shed during the passage of the ghost drifting onto the exposed skin of the observer, which will definitely induce an otherworldly chilling sensation. And: we should keep in mind that some people report that, when they have had a more or less direct contact with a ghost, i.e. they have been directly on its walkabout trajectory, and not been able to jump out of the way, they have experienced a damp, cold sensation as the spirit brushed by, like walking into a heavy cold mist.

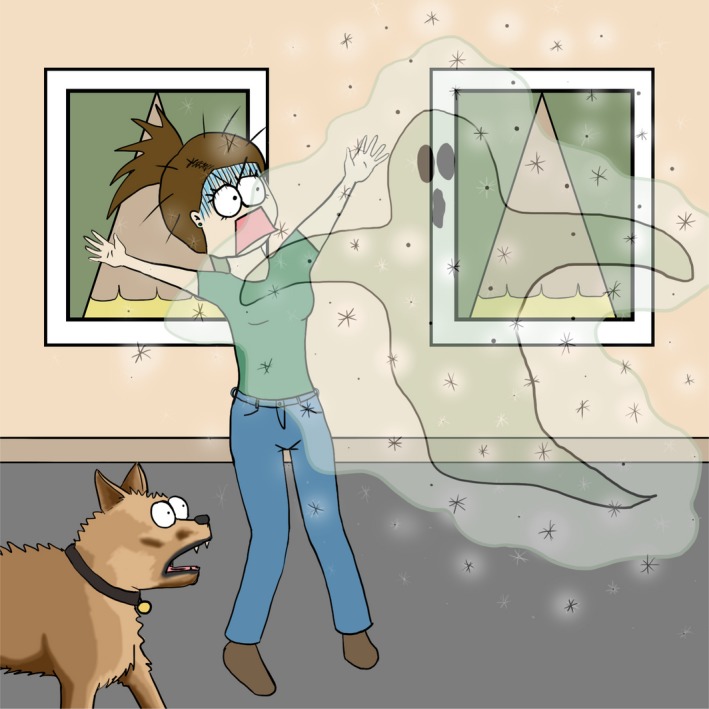

*Ms. Repor‐Tastory, shivering:* Oh: I am beginning to feel the chill right now. I think we better take a short break and a long stiff drink to fortify us for the next part.

## Part 2

*Ms. Repor‐Tastory, slightly flushed:* Welcome back viewers to this absolutely fascinating, if somewhat disturbing topic. But before we return to the story, Ani, tell me: how can you discuss these frightening things in such a relaxed fashion?

*Dr. Noitall‐Most:* Well: I personally do not believe in ghosts, though I have several relatives who do and claim to have actually seen them. But of course, as a scientist, I cannot disprove their existence, either. The thing is: I have never encountered one and, if I do, I do not expect to be harmed by it. How many deaths or injuries caused by ghosts were documented last year? And are we frightened by smoking or driving, despite their associated frightening annual death rates? Have you ever flown to Geneva in Winter and, instead of catching the coach directly to the ski slopes, taken the bus downtown to buy a little frock or bauble for a special après ski, and observed how many people are walking around on crutches, all in name of pleasure/healthy pursuits? No: I do not feel uncomfortable discussing ghosts and their microbiomes.

*Ms. Repor‐Tastory, brightening up:* Gosh, yes, isn't downtown Geneva a paradise for shoppers, especially those who love handbags. I remember one occasion when I was with this incredibly well off broker….(angry noises coming from the in‐ear headphone), oh sorry – you were saying?

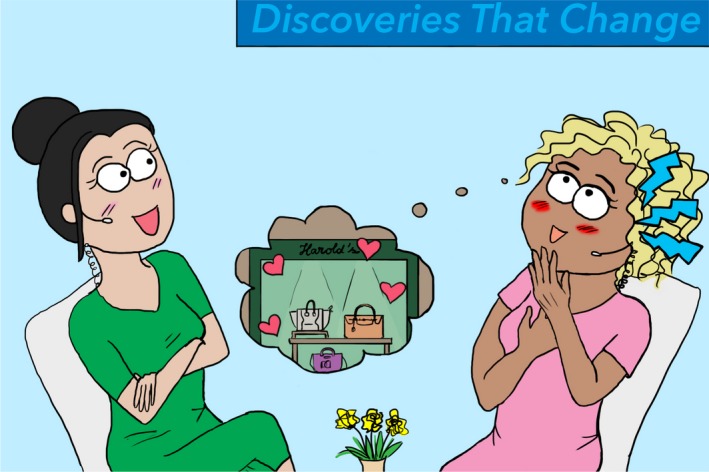

*Dr. Noitall‐Most, dreamily:* Yes, and the fantastic chocolate boutiques…..! But to return to the here and now or, rather, the otherworldly there and then, Mabriella and Ranfredy were frustrated that the only information on GAB was bioinformatic in nature: there was no physiological data. So, after some in‐house brainstorming, they had a nice idea. They isolated individual spores by micromanipulation, using the protocol of Leonie and Hawiovi, and placed them on *GORM* containing radioactive precursors, to allow preliminary assessment of intact metabolic functions, by a new technique called high resolution single cell metabolomics, or *SICMICS*. They then shared with Leonie and Hawiovi their new information, which corroborated a number of the key bioinformatic predictions, and allowed significant refinement of their metabolic models. One important conclusion of the modelling, and now confirmed by the experimental data, was that all GAB lack one or more essential functions for reproduction – they do not have a complete minimal genome – the minimal complement of genes needed for growth and reproduction^30^ – which explains their inability to grow on laboratory media.

*Ms. Repor‐Tastory:* Well, in that case, I do not understand how they exist, if they are not able to perpetuate themselves.

*Dr. Noitall‐Most:* An excellent point, Abi! The thing is that there are many microbes out there that lack essential functions for an autonomous existence: they are called auxotrophs^31^. Pretty much all microbes live in complex communities, the members of which share resources. They practice a complex pattern of division of labour, with different members manufacturing and sharing the vast range of different substances they collectively need for metabolism, growth and reproduction, and thereby economise on use of available resources^31^. So, for example, microbe 1, which receives metabolite X made by microbe 2 in the community, does not need the genetic information to make metabolite X, and may therefore lack this information and thereby save energy by not committing resources to make it. Obviously, microbe 1, which is auxotrophic for X, can grow in its natural environment, but will not grow as a single organism in the laboratory, unless the medium used provides metabolite X.

*Ms. Repor‐Tastory:* Ok, GAB are social beasts, like other microbes – I can understand that – but then another question. If ghost bugs are spores, which are dormant forms and hence inactive, how can they produce the proteins identified by Leonie and Hawiovi, especially those creepy damp chill proteins?

*Dr. Noitall‐Most:* Another excellent question, Abi! This is due to the fact that members of populations of microbes exhibit physiological heterogeneity^32^: they generally exist in a range of different metabolic states, similar to us in fact: while most people go to bed between 10 and 11, go to sleep about 10 min after their heads hit the pillow, and sleep soundly for 7 hours, others watch television until 3 in the morning, or sleep for 4 hours before getting up for a cup of tea and a biscuit, and so on. Even folk belonging to a small, isolated, relatively homogeneous and unchanging community are all a bit different from one another; microbes are the same. Many bugs can live and carry out minimal metabolism with very little food: they have minimal energy/basal power requirements^33^. Heterogeneity of behaviour means, for example, that statistically, at any moment a small percentage of the ghost microbiome would not be dormant spores but rather metabolically‐active vegetative cells and could be expressing INPs on their cell surfaces. At temperatures below 0°C, these would recruit water vapour to form ice crystals, which would subsequently transform to water droplets when temperatures rise above 0°C. These in turn would hydrate neighbouring spores on the dust particles. Traces of food in the air – cooking oil vapour, fish volatiles, etc. in inhabited properties, dust of bat and rodent droppings, etc. in abandoned edifices, would then be sufficient to trigger spore germination and some metabolism, like the production of INPs, fluorescent proteins, gas vesicles, etc. As some spores germinate and undergo limited metabolism, some existing vegetative cells would return to the spore state. This stochastic process ensures that there are always some metabolically active ghost bugs on/in the host.

*Ms. Repor‐Tastory:* I see. So where does all this leave us?

*Dr. Noitall‐Most:* Well, of course, Dr. Geisterbahn was highly excited about the results, and immediately posed the next big question: where do ghost bugs come from? Do ghosts carry part of their old microbiome with them after death of their earthly form, or do they acquire a new, ghostly microbiome?

To answer this, it was necessary to compare ghost microbiomes with original skin microbiomes. This is obviously not a trivial task, but Professor Geisterbahn is an imaginative and energetic soul, and decided to investigate mummies. Important in the selection of study objects was that the bodies of the deceased had not been washed or otherwise treated, so mummies that had been produced by ritual procedures were unsuitable. However, there are a number of examples of naturally mummified bodies not subjected to any known human intervention after death and still wearing the original clothing sported prior to expiration^34^. Crucial, of course, was that any mummy study object had a well‐documented ghost, which drastically reduced the number of possible options, and excluded some interesting study object stratification possibilities. However, after much travel and visits to museums far and wide, particularly neglected ones off the beaten track, Professor Geisterbahn was able to source a handful of mummies that fulfilled the necessary criteria. Serendipitously, a walled‐up 17^th^ century princess had been recently discovered in an abandoned decaying castle in Romania, and was duly recruited into the study cohort. As controls, mummies of individuals thought to have been happy souls, enjoying peaceful, non‐violent deaths, and lacking any reports of post‐death ghostly appearances, were selected.

Anyway, to cut short a long story of a complicated and logistically‐challenging study, comparison of the sporomes of the ambient dust, skin and original clothing remains, and ghost paths, revealed no significant differences between the skin and clothing microbiota of ghostly and non‐ghostly mummies, and no ghostbugs in them, though GAB were readily found in the ghostpaths.

*Ms. Repor‐Tastory:* So where on earth do GAB come from?

*Dr. Noitall‐Most:* Well, Abi: this is of course the 64 million dollar question. If they are not part of the skin microbiota of the original body, they must come from somewhere else: it seems that ghosts, once they decide to become part of our earthly experience, acquire a unique microbiome. But from where? As you know, there have been a number of large scale campaigns to assess and access microbial diversity, and currently there are major international efforts to obtain a comprehensive global inventory^35^. But the thing is this: ghostbugs have never been detected in any of these campaigns, at least thus far.

*Ms. Repor‐Tastory:* So they come out of thin air?

*Dr. Noitall‐Most:* You jest, of course, but in fact this is precisely what Professor Geisterbahn and various members of the paranormal community propose: that in fact the ghost is the link between the world you and I know and love, and another parallel world inhabited by..well. who knows what, but perhaps including the ghostbugs. Of course, microbial ecologists always say: wherever life is possible, microbes will be there. But I am not sure if they would include paranormal habitats in their conceptual frameworks. However, Professor Tim Kennis of the Queenton Institute for Advanced Study has advanced the view that current and past metagenome studies only detect microbes present in samples in numbers above a certain threshold, and that so‐called *rarefaction* 36 assessments of completeness of diversity coverage *only work well when no taxon is extremely rare*, so reliance on them to claim complete diversity coverage is unwarranted. He postulates that there is a fascinating world of as yet undiscovered microbes present in vanishingly small numbers in most environments, the so‐called ultra‐rare biosphere or, perhaps more appropriately in the case of ghostbugs, biological darkest matter^36^, that will only be detected by ultra‐deepest sequencing.

*Ms. Repor‐Tastory:* So, if I understand you correctly, ghostbugs might be everywhere, not just on ghosts?

*Dr. Noitall‐Most:* Yes, this is entirely possible, though pretty much all microbes have preferred habitats or, since they are so small and their immediate environments are also tiny, *microhabitats*. So, even if ghostbugs are everywhere, Professor Geisterbahn would probably argue that their natural habitat is the ghost, and this is where they are to be found in the highest numbers. The thing is: the discovery of new microbes often results from the exploration of new habitats, as was the case with the discovery of ghostbugs. Since some GAB are distantly related to *Gloamingia shiveri*, the vampire bat microbe, the team's current hypothesis is that GAB are ultra‐rare members of communities of the vampire bat microbiome and are right now taking samples from vampire bats and the dust of bat caves at various sites around the world, to analyse by ultra‐deepest metagenomics sequencing.

*Ms. Repor‐Tastory:* Ok, Ani: to make full circle and come back to the exciting research currently taking place on the microbiome of the built environment: what do the experts think of all the ghostly stuff?

*Dr. Noitall‐Most:* Oh, of course, most are highly sceptical. For example, Kennis has stated that, while he considers the results obtained by the German and American groups to be reliable and to constitute a significant advance in accessing and characterising rare microbial diversity, he is totally dismissive of the ghost context and views it as absurd, nothing more than hand waving. He says that, even for those folks who might not discard the possibility of the existence of ghosts, all studies so far are way too preliminary, based on small samples yielding results that do not allow meaningful statistical analysis, and lacking in scientific rigour. Most importantly, they only give correlations and, given the nature of the study objects, the establishment of causalities will be impossible.

*Ms. Repor‐Tastory, looking relieved:* Ok, on that reassuring note, to bring this program to its end, let me pose my usual question: are there any applications emerging from this research?

*Dr. Noitall‐Most:* Yes, Abi, there certainly are, especially in the domain of space travel.

Firstly, efforts to understand what biological functions are essential for life have so far mostly concentrated on analysis of the minimal genome essential for vital activities and reproduction. Now, however, the focus has shifted to what functions are needed for dormancy, since voyages to outer space will take decades, and normal life will not only require impossibly large payloads of food and waste disposal systems, but also result in arrival of very aged if not long‐dead astronauts, or, equally problematic, astronauts completely insane from decades of continually talking about the weather back home, playing Sudoku and repeatedly watching repeats of Top Gear and Big Bang Theory. So the only option is to make them dormant, and the study of the metabolic wherewithal of GAB has now taken centre stage. Interestingly, a number of health companies are also investing heavily in this type of research because they foresee a highly lucrative business in inducing dormancy in, shall‐we‐say, rich‐to‐super rich customers with currently untreatable health issues, until effective cures have been developed. These companies believe that a procedure to induce dormancy will replace the deepfreeze option currently on offer to this type of clientele.

Also in the realm of space travel, there is a lot of activity exploring the radiation‐protection properties of various melanins based on SM, because spacecraft and their occupants are subjected to substantial amounts of hazardous radiation, once out of the Earth's atmosphere^37^. On the commercial scene, a number of personal care companies are investing heavily in SM and SMLAA research to develop next generation sunscreens and more general care products that protect skin against the aging effects of sun and radiation^37^.

*Ms. Repor‐Tastory:* Oh, marvellous! I just love lazing in the sun, but it does wreak havoc with my skin, especially that on the more protruding parts of my body, so a better protectant to those currently available will be a wonderful thing.

*Dr. Noitall‐Most:* Oh, absolutely! And while on the topic of protection, there is an established application relating to ice nucleating bacteria, namely the use of a *non*‐ice‐nucleating bacterium called *Pseudomonas fluorescens* A506 to outcompete, that is, to chase away, ice nucleating bacteria from crop plants and thus reduce frost damage in affected farming regions^38^. It turns out that A506 also outcompetes certain plant disease‐producing microbes, so is also used to prevent fire blight disease of fruit trees^38^, though this is unrelated to the production of INP. This type of ecological pest management will certainly increase in future, as the use of toxic agrochemicals becomes more restricted^39^. Another application of ice nucleating bacteria is in the production of artificial snow^38^. One strain that produces a lot of INP is the basis of a commercial product that, when added to water used in snow makers, enables high quality snow to be produced at higher temperatures. Snowmax^38^ – oh, sorry: I did not mean to name the product to avoid appearing to endorse it – is extensively used in ski resorts in some countries. And, as one might imagine, there are applications in the production of frozen foods, like ice cream, where INP enables the controlled formation of small and stable ice crystals that provide a superior consistency to ice emulsions^38^.

*Ms. Repor‐Tastory:* Wow: this is just amazing!

*Dr. Noitall‐Most:* Yes – without knowing it, we all are exposed in diverse ways to ice nucleating bacteria – they are on the salad plants and frozen foods we consume, the rain that falls on us when we forget the umbrella, the snow we eat after our snowboard clips a rock when we become distracted by thoughts about our competitors at work, ex‐boy/girlfriends, an incoming selfie on the smart phone, and so on.

In terms of other applications of ghostbug research, two very exciting synthetic biology projects are currently underway at the Lorenzo von Syntech High Security Institute for Artificial Life in Madrid, headed by the world‐renowned Professor Vic Torde. One is to create a *Bacillus subtilis* cell factory for the high‐level production of SM, and variants of this melanin, which can be applied as sort of radioprotective *skin* for coating spacecraft and for incorporating into astronaut accoutrements^37^. An even more interesting goal is to introduce into GAB strains the missing genes present in minimal genomes of bugs able to grow and reproduce, in order to create GAB derivatives that can be grown and properly studied in the laboratory. Comparison of GAB with their viable, vital variants will advance our understanding of dormancy and longevity, and how it can be exploited in various organisms, including us.

*Ms. Repor‐Tastory:* But isn't there a possibility that a microbial Frankenstein will be created?

*Dr. Noitall‐Most:* Good question, Abi! In fact, Vic is very conscious of this possibility and his colleagues will therefore simultaneously engineer into GAB study objects conditional lethal genes that will only allow the new bugs to survive under special, restrictive laboratory conditions^40^.

*Ms. Repor‐Tastory:* Well, I have the feeling that this program will fuel a lot of thought and discussion. The possible existence of ghosts is obviously a matter more contentious now than before; I guess the microbes, as always, are keeping all options open. In the meantime, thank you, Ani, for this highly interesting expose, and thank you viewers for joining us for this episode of ‘*Microbiome Discoveries that Change our Lives*’.

## Conflict of interest

None declared.

## Explanatory Notes for Online Version at http://www.theabsurdmicrobe.com

5 youngest daughter of the renowned Dr. Noitall, see e.g. Koshland, D.E., Jr. (1994) Editorial: A simple proposal for health care rationing. *Science* **264**: 1515

6 Lax, S., Smith, D.P., Hampton‐Marcell, J., et al. (2014). Longitudinal analysis of microbial interaction between humans and the indoor environment. *Science* **345**:1048‐52

7 Kellogg, C. A. and Griffin, D. W. 2006. Aerobiology and the global transport of desert dust. *Trends Ecol. Evol*. **21**: 638‐644

8 Sender, R., Fuchs, S., and Milo, R. (2016) Are We Really Vastly Outnumbered? Revisiting the Ratio of Bacterial to Host Cells in Humans. *Cell* **164**: 337–340; Qian, J., Hospodsky, D., Yamamoto, N., Nazaroff, W.W. and Peccia, J. (2012) Size‐resolved emission rates of airborne bacteria and fungi in an occupied classroom. *Indoor Air* **22**: 339–351; Hospodsky, D., Yamamoto, N., Nazaroff, W.W., et al. (2015) Characterizing airborne fungal and bacterial concentrations and emission rates in six occupied children's classrooms. *Indoor Air* **25**: 641–52; Jaffal, A.A., Banat, I.M., El Mogheth, A.A., et al. (1997) Residential indoor airborne microbial populations in the United Arab Emirates. *Environm. Int*. **23**: 529–533.

9 Hampton‐Marcell, J.T., Lopez, J.V. and Gilbert, J.A. (2017) The human microbiome: an emerging tool in forensics. *Microb Biotechnol*. **10**: 228‐230.

10
https://www.theatlantic.com/magazine/archive/2016/06/a-reasonable-doubt/480747/; https://www.theguardian.com/science/2017/oct/02/dna-in-the-dock-how-flawed-techniques-send-innocent-people-to-prison

11 Lax, S., Nagler, C.R. and Gilbert, J.A. (2015) Our interface with the built environment: immunity and the indoor microbiota. *Trends Immunol*. **36:** 121‐3; Gilbert, J.A. (2017) How do we make indoor environments and healthcare settings healthier? *Microb Biotechnol*. **10**:11‐13;

12
https://en.wikipedia.org/wiki/Bureau_for_Paranormal_Research_and_Defense

13 Romero‐Tabarez, M., Jansen, R., Sylla, M. et al. (2006) 7‐O‐malonyl macrolactin A, a new macrolactin antibiotic from *Bacillus subtilis* active against methicillin‐resistant *Staphylococcus aureus*, vancomycin‐resistant enterococci, and a small‐colony variant of *Burkholderia cepacia*. *Antimicrob Agents Chemother*. **50**: 1701‐9; Calle, F. (2017) Editorial: The microbiome as a source of new enterprises and job creation: Marine microbiome as source of natural products. *Microb. Biotechnol*. **10**: 1293‐1296

14 Timmis, K., Jebok, F., Molinari, G. and Rohde, M. (2017) Microbiome yarns: microbiome basis of memory. *Microb. Biotech*. **10**: 1283‐1292.

15 No, sorry: this is not an image of ghostspores present on a ghostpad, but rather a collage of images of diverse spore types produced by aerial mycelia of different strains of Actinomycete

16 Marcy, Y., et al (2007) Dissecting biological “dark matter” with single‐cell genetic analysis of rare and uncultivated TM7 microbes from the human mouth. *Proc Natl Acad Sci U S A*. **104**: 11889–11894; Stepanauskas, R. (2012) Single cell genomics: an individual look at microbes. *Curr. Op. Microbiol*. **15**: 613‐620; Woyke, T. and Jarett, J. Function‐driven single‐cell genomics (2015) *Microb. Biotechnol*. **8**: 38–39.

17 Helfrich, E.J.N., Reiter, S. and Piel, J. (2014) Recent advances in genome‐based polyketide discovery. *Cur. Op. Biotechnol*. **29**: 107‐115; Lackner, G., Peters, E., Helfrich, E.J.N and Piel, J. (2017) Insights into the lifestyle of uncultured bacterial natural product factories associated with marine sponges *Proc. Natl. Acad. Sci*. USA **114**: E347‐E356; Mori T., Cahn J., Wilson M. et al. (2018) Single‐bacterial genomics validates rich and varied specialized metabolism of uncultivated *Entotheonella* sponge symbionts. *Proc. Natl. Acad. Sci. USA* **115**: 1718‐1723.

18 Native American Indian male name meaning *Going down the ladder* (http://www.sheknows.com/baby-names/name/hawiovi)

19 No sorry, these are not ghostbacteria, but different cell types of strains isolated by Ken Timmis, Joan Timmis and Balbina Nogales from the phylospheres of gorgeous plants on the beautiful campus of the University of the Balearic Isles, following a sampling (vegetation denuding) campaign, while KT was a short‐term postdoc of BN, to whom he expresses infinite gratitude, and to the UIB for a grant supporting his stay in Palma. Back in Braunschweig, Gabriella Molinari and Manfred Rohde revealed the fascinating ultrastructures of isolate 2H5 (images 1, 3 and 4), from *Populus nigra*, which was identified as a *Hymenobacter* sp., and isolate 2A1 (images 2, 5 and 6), from a cactus flower, which was identified as a strain of *Hymenobacter ocellatus*, the type strain of which is, however, distinctly less ghostly and altogether quite boring in appearance. The spores in the image are conidia from *Aspergillus fumigatus*.

20 The naming of *Spookia bogeymana* provoked a certain amount of controversy, because a newish member of the International Committee on Systematics of Prokaryotes objected on the grounds that the species name was sexist and, since members of this genus came from ghosts of both sexes, they should be named *S. bogeyhumana*. According to one insider, the discussion became rather heated and lines were drawn, not as usual between (c)lumpers and splitters (Love, M. (1982) A rose by any other name. New Sci. 94: 431‐2; Endersby, J. (2009) Lumpers and splitters: Darwin, Hooker, and the search for order. Science. **326**: 1496‐9), but rather between younger (politically correct) and older (Benny Hill‐Monty Python‐raised) members. Eventually, the majority of the members of the Committee, which has an average age of 68, concluded that the disputed term *bogeyman* was a generic term for (ostensibly imaginary) monsters used to frighten children into good behaviour, and thus the gender of the ghost was a red herring; the Committee allowed the proposed name.

21 Since almost all of the bacterial sporeforming ghostbugs were related to one another, but not to other known bacteria, they were assigned to a new phylum: the Candidate Division Spookybacteria.

22 see, e.g. Schneiker, S., Martins dos Santos, V.A., Bartels, D. et al. (2006) Genome sequence of the ubiquitous hydrocarbon‐degrading marine bacterium *Alcanivorax borkumensis. Nat Biotechnol*. **24**: 997‐1004.

23 Klebahn, H. (1895) Gasvakuolen, ein Bestandteil der Zellen der wasserblütenbildenden Phycochromaceen. *Flora* **80**: 241–282; Walsby, A.E. (1994) Gas vesicles. *Microbiol Rev*. **58**: 94–144; Pfeifer, F. (2012) Distribution, formation and regulation of gas vesicles. *Nat Rev Microbiol*. **10:** 705‐15.

24 Tsien, R.Y. (1998). The green fluorescent protein. *Annu. Rev. Biochem*. **67**: 509–44; Rodriguez, E.A., Tran, G.N., Gross, L.A., et al.* *(2016). A far‐red fluorescent protein evolved from a cyanobacterial phycobiliprotein. *Nature Methods*. **13**: 763–9; Rodriguez, E.A., Campbell, R.E., Lin, J.Y., et al. (2017). The Growing and Glowing Toolbox of Fluorescent and Photoactive Proteins. *Trends Biochem. Sci*. **42**: 111–129.

25 Chalfie, M., Tu, Y., Euskirchen, G., et al. (1994). Green fluorescent protein as a marker for gene expression. *Science* **263**: 802–5; Suarez, A., Güttler, A., Strätz, et al. (1997) Green fluorescent protein‐based reporter systems for genetic analysis of bacteria including monocopy applications. Gene **196**: 69‐74; Stepanenko, O.V., Verkhusha, V.V.,* *Kuznetsova, I.M., et al (2008)*. *Fluorescent proteins as biomarkers and biosensors: throwing color lights on molecular and cellular processes. *Curr. Prot. Pept. Sci*. **9**: 338–69.

26 Solano, F. (2014) Melanins: Skin Pigments and Much More—Types, Structural Models, Biological Functions, and Formation Routes. *New J. Sci*. **2014**: 498276; Cordero, R.J.B. and Casadevall A. (2017) Functions of fungal melanin beyond virulence. *Fungal Biol Rev* **31**: 99–112; Zeng, Z., Guo, X.P., Cai, X., et al. (2017) Pyomelanin from *Pseudoalteromonas lipolytica* reduces biofouling. *Microb Biotechnol*. **10**: 1718‐1731; Schmaler‐Ripcke, J., Sugareva, V., Gebhardt, P. et al. (2009) Production of pyomelanin, a second type of melanin, via the tyrosine degradation pathway in *Aspergillus fumigatus*. *Appl Environ Microbiol* 75: 493–503; Rodriguez‐Rojas, A., Mena, A., Martin, S. et al. (2009) Inactivation of the *hmgA* gene of *Pseudomonas aeruginosa* leads to pyomelanin hyperproduction, stress resistance and increased persistence in chronic lung infection. *Microbiol‐Sgm* **155**: 1050–1057.

27 Oren, A. and Gunde‐Cimerman, N. (2007) Mycosporines and mycosporine‐like amino acids: UV protectants or multipurpose secondary metabolites? *FEMS Microbiol. Lett*. **269**: 1–10; Kicklighter, C.E., Kamio, M., Nguyen, L., Germann, M.W. and Derby, C.D. (2011) Mycosporine‐like amino acids are multifunctional molecules in sea hares and their marine community. *Proc. Natl. Acad. Sci. USA* **108**: 11494‐11499; there are quite a few variations on the term *black as Newgate's knocker*: see, e.g. http://www.worldwidewords.org/qa/qa-bla4.htm

28
https://en.wikipedia.org/wiki/Ectoplasm_(paranormal); https://www.theepochtimes.com/ectoplasm-ghost-slime-seriously-studied-by-a-nobel-prize-laureate-and-other-scientists_862611.html

29 Arny, D.C., Lindow, S.E. and Upper, C.D. (1976) Frost sensitivity of *Zea Mays* increased by application of *Pseudomonas syringae*. *Nature* **262**: 282‐284; Lindow, S.E., Arny, D.C. and Upper, C.D. (1982) Bacterial ice nucleation: a factor in frost injury to plants. *Plant Physiol*. **70**: 1084‐9; Gurian‐Sherman, D and Lindow, S.E. (1993) Bacterial ice nucleation: significance and molecular basis. *FASEB J*. **14**: 1338‐43.

30 Akerley, B.J., Rubin, E.J. Camilli, A. et al. (1998). Systematic identification of essential genes by in vitro mariner mutagenesis. *Proc. Natl. Acad. Sci*. *USA*. **95**: 8927–32; Hutchison III, C.A., Peterson, S.N, Gill, S.R., et al (1999). Global transposon mutagenesis and a minimal *Mycoplasma* genome. *Science*. **286**: 2165–9; Hutchison III, C.A., Chuang, R.Y., Noskov, V.N., et al. (2016) Design and synthesis of a minimal bacterial genome. *Science* **351:** 1414‐

31 e.g. see Droop, M.R. (1957) Auxotrophy and organic compounds in the nutrition of marine phytoplankton. *J. Gen. Microbiol*. **16**: 286‐293; Helliwell, K.E., Wheeler, G.L. and Smith, A.G. (2013) Widespread decay of vitamin‐related pathways: coincidence or consequence? *Trends Genet*. **29**: 469‐478; Pelz, O., Tesar, M., Frech, G., et al. (1999) Towards elucidation of microbial community metabolic pathways: unravelling the network of carbon sharing in a pollutant‐degrading bacterial consortium by immunocapture and isotopic ratio mass spectrometry. *Environm. Microbiol*., **1**: 167‐174.

32 Spudich, J.L and Koshland Jr, D.E. (1976) Non‐genetic individuality: chance in the single cell. *Nature* **262**: 467‐471; Colman‐Lerner, A., Gordon, A., Serra, E., et al. (2005) Regulated cell‐to‐cell variation in a cell‐fate decision system. *Nature* **437** : 699–706; Smits, W.K., Kuipers, O.P. and JW Veening, J.W. (2006) Phenotypic variation in bacteria: the role of feedback regulation. *Nature Reviews Microbiology* **4**: 259–271

33 Hoehler, T.M. and Jørgensen, B.B. (2013). Microbial life under extreme energy limitation. *Nat. Rev. Microbiol*. **11**, 83–94; Lever, M.A., Rogers, K.L., Lloyd, K.G., et al. (2015). Life under extreme energy limitation: a synthesis of laboratory‐and field‐based investigations. *FEMS Microbiol. Rev*.** 39**, 688–728; Orsi, W.D., Richards, T.A. and Santoro A.E. (2015). Cellular maintenance processes that potentially underpin the survival of subseafloor fungi over geological timescales. *Estuarine Coast. Shelf Sci*. **164**, A1–A9.

34
http://www.momtastic.com/webecoist/2013/02/01/most-intriguing-mummies-perfectly-preserved-human-bodies/; https://news.nationalgeographic.com/news/2007/09/photogalleries/mummy-pictures/

35 Venter, J.C., Remington, K., Heidelberg, J.F., et al. (2004) Environmental genome shotgun sequencing of the Sargasso Sea. *Science* **304**: 66–74; Alivisatos, A.P., Blaser, M.J., Brodie, E.L., et al. (2015) MICROBIOME. A unified initiative to harness Earth's microbiomes. *Science* **350**: 507‐8; Salazar, G., Cornejo‐Castillo, F.M., Benitez‐Barrios, V. et al. (2016) Global diversity and biogeography of deep‐sea pelagic prokaryotes. *ISME J* **10**: 596–608; Thompson, L. R., Sanders, J.G., McDonald, D, et al. (2017) A communal catalogue reveals Earth's multiscale microbial diversity. *Nature* **551**: 457‐463.

36 Locey, K.J. and Lennon, J.T. (2016) Scaling laws predict global microbial diversity. Proc. Natl. Acad. Sci. USA. 113: 5970‐5; Sanders, H.L. (1968) Marine benthic diversity: a comparative study. *Amer. Naturalist* **102**: 243‐282; Gotelli, N.J., Colwell, R.K., Magurran, A.E. and McGill, B.J. (2011) Estimating species richness. *Frontiers in Measuring Biodiversity*. Oxford University Press, New York 39‐54; Sogin, M.L., Morrison, H.G., Huber, J.A. et al. (2006) Microbial diversity in the deep sea and the underexplored “rare biosphere”. *Proc. Natl. Acad. Sci. USA*. **103**: 12115‐12120; Marcy, Y., Ouverney, C. Bik, E.M. et al. (2007) Dissecting biological “dark matter” with single‐cell genetic analysis of rare and uncultivated TM7 microbes from the human mouth. *Proc. Natl. Acad. Sci. USA*. **104**: 11889‐11894; Robbins, R.J., Krishtalka, L. and Wooley, J.C. (2016) Advances in biodiversity: metagenomics and the unveiling of biological dark matter. *Stand Genomic Sci*. **11**: 69.

37 Lagunas‐Munoz, V.H., Cabrera‐Valladares, N., Bolivar, F., Gosset, G., and Martinez, A. (2006) Optimum melanin production using recombinant *Escherichia coli*. *J Appl Microbiol* 101: 1002–1008; Cordero, R.J.B. (2017) Melanin for space travel radioprotection. *Environm. Microbiol*. **19**: 2529–2532; Cordero, R.J.B., Vij, R and Casadevall, A. (2017) Microbial melanins for radioprotection and bioremediation. *Microb Biotechnol*. **10**: 1186–1190; Chrapusta, E., Kaminski, A., Kornelia Duchnik, K. et al. (2017) Mycosporine‐like amino acids: potential health and beauty ingredients. *Mar Drugs*. **15**: 326.

38 Lindow, S.E. (1987) Competitive exclusion of epiphytic bacteria by Ice^−^ *Pseudomonas syringae* mutants. *Appl. Environ. Microbiol*. **53:** 2520‐2527; Lindow, S.E. (1995) Control of epiphytic ice nucleation‐active bacteria for management of plant frost injury, p. 239‐256. *In* Lee, R.E., Warren, G.J. and Gusta, L.V. (eds.), *Biological Ice Nucleation and its Applications*. APS Press, St. Paul, Minn.; http://www.nufarm.com/USAg/BlightBanrA506 ; Johnson, K. B., and Stockwell, V.O. (1998) Management of fire blight: a case study in microbial ecology. *Annu. Rev. Phytopathol*. **36:** 227‐248; Mercier, J., and Lindow, S.E. (2001) Field performance of antagonistic bacteria identified in a novel laboratory assay for biological control of fire blight of pear. *Biol. Control* **22:** 66‐71; Stockwell, V.O., Johnson, K.B., Sugar, D., and Loper, J.E. (2010) Control of fire blight by *Pseudomonas fluorescens* A506 and *Pantoea vagans* C9‐1 applied as single strains and mixed inocula. *Phytopathol*. **100**: 1330‐9; http://www.snomax.com; Cid, F.P., Rilling, J.I., Graether, S.P., et al. (2016) Properties and biotechnological applications of ice‐binding proteins in bacteria. *FEMS Microbiol Lett*. **363** https://doi.org/10.1093/femsle/fnw099; https://www.youtube.com/watch?v=SenJud3cHLc

39 see, e.g. Wackett, L.P. (2013) Microbial control agents. An annotated selection of World Wide Web sites relevant to the topics of Microbial Biotechnology. *Microb. Biotechnol*. **6:** 443‐444; Mitter, B., Pfaffenbichler, N. and Sessitsch, A. (2016) Plant‐microbe partnerships in 2020. *Microb. Biotechnol*. **9:** 635‐640.

40 e.g. Munthali, M.T., Timmis, K.N. and Diaz, E. (1996) Restricting recombinant DNA dispersal: design of a contained biological catalyst. *Bio/Technology* **14**: 189‐191; Torres, B., Jaenecke, S., Timmis, K.N., Garcia, J and Diaz, E. (2003) A dual lethal system to enhance containment of recombinant micro‐organisms. Microbiology **149**: 3595‐3601.

